# Correction: Chang et al. Intertypic Recombination Between Coxsackievirus A16 and Enterovirus A71 Structural and Non-Structural Genes Modulates Virulence and Protection Efficacy. *Vaccines* 2025, *13*, 1017

**DOI:** 10.3390/vaccines14020137

**Published:** 2026-01-29

**Authors:** Hooi Yee Chang, Han Kang Tee, Kien Chai Ong, Kartini Jasni, Syahril Abdullah, I.-Ching Sam, Yoke Fun Chan

**Affiliations:** 1 Department of Medical Microbiology, Faculty of Medicine, Universiti Malaya, Kuala Lumpur 50603, Malaysia; hooiyee@um.edu.my (H.Y.C.); jicsam@ummc.edu.my (I.-C.S.); 2 Department of Microbiology and Molecular Medicine, University of Geneva, 1211 Geneva, Switzerland; han.tee@unige.ch; 3 Department of Biomedical Sciences, Faculty of Medicine, Universiti Malaya, Kuala Lumpur 50603, Malaysia; kcong@um.edu.my; 4 Comparative Medicine and Technology Unit, Institute of Bioscience, Universiti Putra Malaysia, Serdang 43400, Malaysia; kartini.jasni@upm.edu.my; 5 Department of Biomedical Sciences, Faculty of Medicine and Health Sciences, Universiti Putra Malaysia, Serdang 43400, Malaysia; syahril@upm.edu.my; 6 Malaysia Genome and Vaccine Institute, National Institutes of Biotechnology Malaysia, Kajang 43000, Malaysia

In the original publication [1], there was a mistake in Figure 2. The names of all the chimeras, Chi-CCE, Chi-ECE, Chi-EEC, and Ch-CEC, are wrong and have been corrected in Figure 2A,B. The correct Figure 2 and legend appear below.

The authors state that the scientific conclusions are unaffected. This correction was approved by the Academic Editor. The original publication has also been updated.

## Figures and Tables

**Figure 2:**
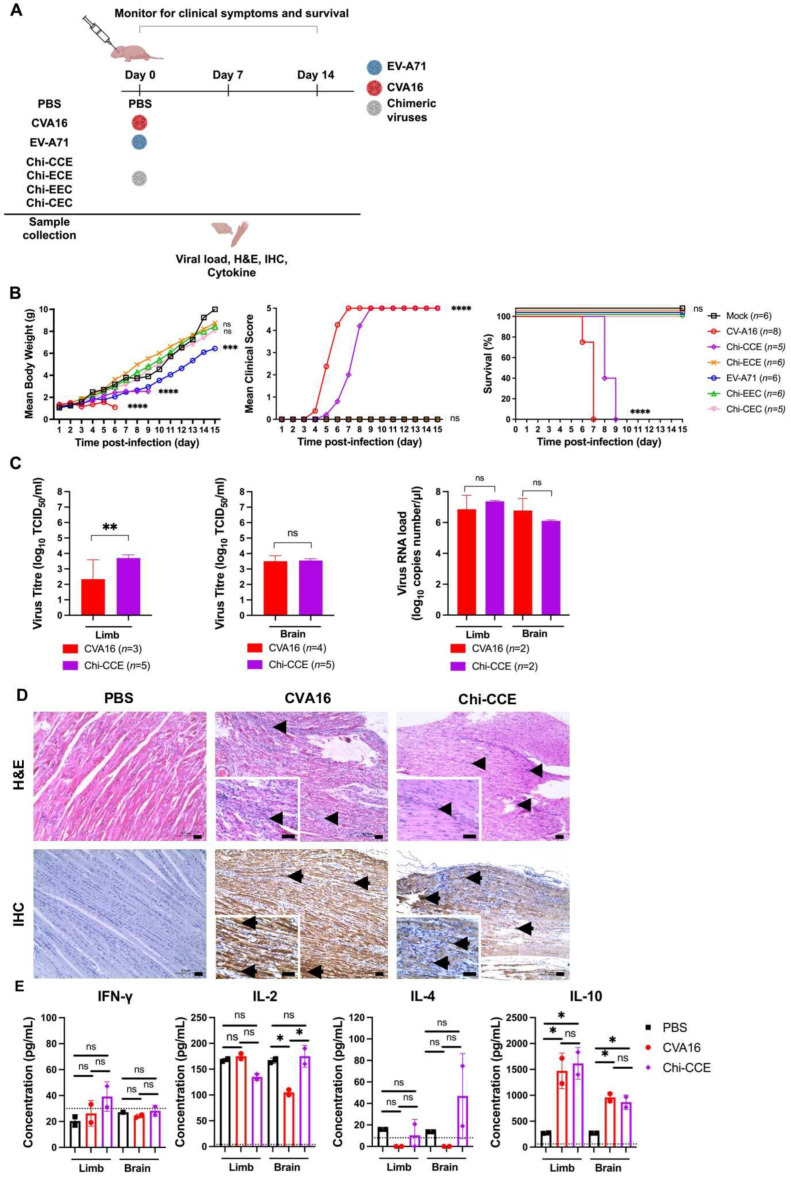
Virulence of chimeric viruses in mice. (**A**) Diagram illustrating the *in vivo* experimental design used to assess chimeric virus virulence. (**B**) Mean body weight, clinical score, and survival rate of infected mice were monitored, with the number of mice indicated as *n*. Chimeric virus-infected mice were compared with PBS-inoculated mice. (**C**) Viral titration (log_10_ TCID_50_/mL) using TCID_50_ assay and virus RNA load (log_10_ copies/µL) using real-time qPCR in brains and limbs. The data are presented as mean ± SD (*n* > 2). Viral titre and viral RNA load in Chi-CCE-infected mice were compared with CVA16-infected mice. (**D**) H&E staining and IHC analysis for PBS-inoculated, CVA16-infected, and Chi-CCE-infected mice. Arrowheads indicate inflammatory cell infiltrates, while arrows indicate viral antigens. Scale bar: 50 µm. Magnification: 10× and 40× (small box). (**E**) The concentrations of cytokines including IFN-γ, IL-2, IL-4, and IL-10 in the sample supernatants were measured by ELISA. Dotted lines indicate limit of detection for the ELISA. Results are presented as mean ± SD (*n* = 2). Statistically significant differences after Bonferroni correction are denoted with * *p* < 0.05, ** *p* < 0.01, *** *p* < 0.001, **** *p* < 0.0001, and no significant (ns).
