# Supplementary material for: Intertypic Recombination Between Coxsackievirus A16 and Enterovirus A71 Structural and Non-Structural Genes Modulates Virulence and Protection Efficacy
Source: Vaccines (Basel). 2025 Sep 29;13(10):1017. doi: 10.3390/vaccines13101017 (PMC12567581; doi:10.3390/vaccines13101017)
Supplement: Supplementary file 1 [file vaccines-13-01017-s001.zip › vaccines-3865591-supplementary.pdf]

Table S1. Primers used in EMP PCR to construct chimeric clones.

Primer set	Target gene	Sequences (5′-3′)	Genome location	1st annealing temperature (°C)	2nd annealing temperature (°C)
CV P2 F (F1)	CVA16 NS	GGAAAGTTTGGGCAGCAATCAGGTG	3332-3356 (CVA16)	72	72
EV71 NS chi EMP R1	EV-A71 /CVA16 NS	GGGGTTCAGCTTTAAACTGTAATATTAAAAT AATTCGAGCCAATTTCTTC	7303-7352 (EV-A71) 7302-7350 (CVA16)		
EV71 NS chi EMP R2	EV-A71 VP1	AAGGGTAGTAATGGCAGTACGACTAGTGCC G	3302-3332 (EV-A71)		
CV ATG P1 F	CVA16 VP4	ATGGGGTCACAAGTCTCCACTCAGCGTTCC	746-775 (CVA16)	72	66
EV (CV P1) EMP R1	CVA16 VP1	AGATTGCTGGCCGAACTTTCCCAAAGTTGTT ATTTTGTCTCTACT	3333-3353 (EV-A71) 3308-3331(CVA16)		
EV (CV P1) EMP R2	EV-A71 5 UTR	TTCTCACAATTAAGGAGTAATATATAATCAA GAGT	712-746 (EV-A71)		

Table S2. Primers used in colony PCR screening and DNA sequencing.

Primers	Target gene	Sequences (5′-3′)	Genome location	Nucleotide length (bp)
EV1.1F	CVA16 VP1	CACATTTGTCGTAGCCAAAC	2845-2864	1220
EV1.2R	EV-A71 NS	TGGGGATACCTAAAATGGATG	4045-4065	
CV1.1F	CVA16 VP3	GAGGTTAACAACCTGAAGAC	1874-1893	2523
CV1.2R	EV-A71 NS	CTTCTCTAGAACATAGACTCG	4377-4397	
Ep5F	EV-A71 VP1	AGGAGAGATAGATCTCCCTCTTGA	2714-2734	1168
CP5R	CVA16 NS	TAAATGGTTCTTCAACGCCTCC	3856-3877	
